# Hydrophilic Sponges for Leaf‐Inspired Continuous Pumping of Liquids

**DOI:** 10.1002/advs.201700028

**Published:** 2017-04-19

**Authors:** Tingjiao Zhou, Jinbin Yang, Deyong Zhu, Jieyao Zheng, Stephan Handschuh‐Wang, Xiaohu Zhou, Junmin Zhang, Yizhen Liu, Zhou Liu, Chuanxin He, Xuechang Zhou

**Affiliations:** ^1^ College of Chemistry and Environmental EngineeringShenzhen UniversityShenzhen518060P. R. China; ^2^ Department of ChemistryThe Chinese University of Hong KongShatin N.THong Kong SARP. R. China

**Keywords:** elastic sponges, liquid transport, microfluidics, pumping, surface modification

## Abstract

A bio‐inspired, leaf‐like pumping strategy by mimicking the transpiration process through leaves is developed for autonomous and continuous liquid transport enabled by durable hydrophilic sponges. Without any external power sources, flows are continuously generated ascribed to the combination of capillary wicking and evaporation of water. To validate this method, durable hydrophilic polydimethylsiloxane sponges modified with polyvinyl alcohol via a “dip‐coat‐dry” method have been fabricated, which maintains hydrophilicity more than 2 months. The as‐made sponges are further applied to achieve stable laminar flow patterns, chemical gradients, and “stop‐flow” manipulation of the flow in microfluidic devices. More importantly, the ease‐of‐operation and excellent pumping capacity have also been verified with over 24 h's pumping and quasi‐stable high flow rates up to 15 µL min^−1^. The present strategy can be easily integrated to other miniaturized systems requiring pressure‐driven flow and should have potential applications, such as cell culture, micromixing, and continuous flow reaction.

Highly defined liquid transport is an important issue in both nature and academic fields as well as practical applications in chemistry, physics, biology, energy, and engineering.^1^, ^2^, ^3^, ^4^, ^5^, ^6^, ^7^, ^8^, ^9^, ^10^, ^11^, ^12^ Natural living systems have adapted to function in the best energy‐saving way, among which transpiration is one good example of the autonomous ascent of water in plants. For instance, *eucalyptus regnans*, the tallest of all flowering plants, elevates the needed water and nutrients as high as over 100 m and a large oak tree transpires totally ≈151 kL year^−1^. Drawing inspiration from nature, the copious quantity of attention for such automatic and self‐powered systems has been drawn in the scientific community toward liquid capture,^13^, ^14^, ^15^, ^16^, ^17^, ^18^ liquid delivery,^19^, ^20^, ^21^, ^22^, ^23^, ^24^ phase separation,^25^ and so forth. Specifically, Cao et al.^13^ fabricated an artificial fog collector through integrating cactus spine‐like hydrophobic conical microtip arrays with the hydrophilic cotton matrix for efficient fog water collection, transportation and preservation, the strategy of which functioned as well for the continuous and effective collection of micron‐sized oil droplets in oil–water–solid three‐phase systems underwater.^25^ Another intriguing example is mimicking of wetted spider silk.^18^ The unique fibre structure with periodic spindle‐knots and joints resulted in continuous condensation and directional collection of water drops. Such fibrous system for controlled liquid transfer was further investigated in detail by using Chinese brushes with the demonstration of directly writing of micro‐lines via parallel hairs.^24^ To achieve dynamic manipulation of water droplets, a humidity responsive switch was prepared through the integration of polyacrylic acid (PAA) hydrogel, cellulose membrane and two surface modified copper wires.^21^ The open–close motion of the switch, which was in relation to the swelling or shrinking state of PAA hydrogel triggered by humidity changes, propelled tiny water droplet sliding along the copper wires directionally and efficiently. However, attempts on these artificial systems often suffer from sophisticated designs and complex fabrication procedures, albeit with their excellent performance.

For the abovementioned examples, the driving force typically originates from the gradient of Laplace pressure, capillary force, or/and wettability difference on 1D or 2D materials. Consequently, surfaces with nano/microscale hierarchical structures and special wetting properties are highly demanded. Interestingly, a superhydrophobic pump, composing a thin porous superhydrophobic mesh and a polymer tube was established successfully for continuous and spontaneous antigravity water ascent up to a centimeter height scale without any external forces.^26^ The driving force arises only from the surface energy released upon coalescence between the droplet and the water column. More studies utilizing 3D porous structures for directional liquid transport have also been demonstrated very recently.^27^, ^28^, ^29^, ^30^ Even so, smart methods for controlled liquid transport systems, possessing the features of ease‐of‐operation, portability, and robust and excellent performance, are needed to expand their application scope.

To further advance this field, we developed a strategy that combines the capillary action and evaporation of water to mimic the continuous pumping in biosystem. To achieve such leaf‐like continuous pumping of water, the system requires a long‐term hydrophilic modification of surfaces and 3D‐interconnected porous structures. Notably, polydimethylsiloxane (PDMS) sponges are stretchable and compressible structures made of elastomeric materials, which can be fabricated by a cost‐effective sugar leaching method. Owing to their unique mechanical, chemical and physical properties, in our recent studies, PDMS sponges were severed as 3D elastic skeletons for the high‐performance of 3D stretchable and compressible conductors,^31^ all‐soft conductors,^32^ oil/water separation interfaces,^33^ and organic sponge photocatalyst.^34^ In this study, PDMS sponges provide microchannel networks for the fast and efficient liquid transportation. “Dip‐coat‐dry” modification of the PDMS sponge surface using polyvinyl alcohol (PVA) was carried out to attain long‐term hydrophilicity for water wicking by capillary action. Consequently, water continuously evaporates through all pores at the PVA‐PDMS sponge/air interface. To validate this strategy, we fabricated and modified the PDMS sponges to obtain long‐term preservation of the hydrophilicity, which lasted for several weeks. We further applied the as‐made hydrophilic sponges to generate chemical gradients and manipulate flows. A flow rate of up to 15 µL min^−1^ was obtained and well‐defined laminar flow patterns were achieved and nourished for more than 24 h owing to water evaporation.

The fabrication and surface modification of the hydrophilic PVA‐PDMS sponges are illustrated in **Figure** **1**a. First, PDMS sponges with a typical size of 18 × 17 × 9 mm^3^ were fabricated by using a cost‐effective sugar leaching method as described in our previous work.^31^, ^32^ Briefly, Sugar cubes as templates were immersed in the mixture of PDMS prepolymer and curing agent at a weight ratio of 10:1, followed by degassing for 2 h. PDMS on the surface of cubes was wiped off to expose the sugar after curing in a 60 °C oven for 3 h. The sugar template was then dissolved in a hot water bath (60 °C) and washed away under stirring. After drying at 60 °C for 10 h, PDMS sponges were obtained. Thereafter, the surface modification of PDMS sponges was conducted by applying a “dip‐coat‐dry” method.^35^ During this process, the as‐made PDMS sponges were first treated with air plasma, then dipped in 1 wt% PVA solution for 20 min and dried at 65 °C. In order to get a uniform modification of the PVA, the dip‐coat‐dry process was repeated for five times, the adsorbed PVA at the PDMS surface was heat‐immobilized at 110 °C to obtain hydrophilic PVA‐PDMS sponges.

**Figure 1:**
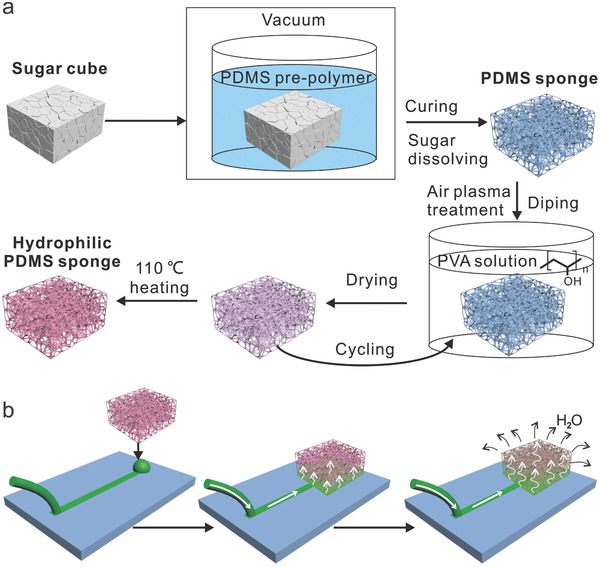
a) Schematic illustration of the fabrication processes of hydrophilic PDMS sponges by sugar leaching method and subsequent “dip‐coat‐dry” method. b) Schematic illustration of the leaf‐like, pumping mechanism using coupled capillary force and water evaporation by applying the hydrophilic PVA‐PDMS sponges.

The cartoon in Figure 1b shows the working mechanism of the as‐made hydrophilic PVA‐PDMS sponges. Initially, the microchannels were filled up with liquid. When the hydrophilic sponge comes into contact with the water at the outlet, capillary force‐driven pumping starts to generate continuous flows as the inlets are connected to solution reservoirs. Meanwhile, water evaporates through the micropores of the sponge exposed to air and ultimately the whole setup achieves a constant evaporation rate after the sponge is fully loaded with water. A “non‐stop” flow is established as long as water evaporates through the hydrophilic sponge surfaces.

**Figure** **2**a shows optical images of the original sugar cube, PDMS sponge, air‐plasma treated PDMS sponge and PVA‐PDMS sponge. Figure 2b,c shows the porous morphology of the as‐made PDMS sponges and of PDMS sponges after surface modification with PVA solution. To evaluate the wettability of the sponges, the absorption time of water (2 µL) is applied as a parameter. The absorption time describes how fast a water droplet is absorbed into the PDMS sponges. To guarantee a uniform coating of the PVA, multicycles of the dip‐coat‐dry process were conducted. Indeed, the absorption time decreases dramatically from ≈80 to 14 s with increasing coating cycles from 1 to 5. Figure 2e shows the as‐made PVA‐PDMS sponges can still absorb water droplet even after a storage time of more than 2 months with increasing absorption time, which may be related to unspecific adsorption of molecules to the sponge surface. Without PVA coating, the hydrophobicity of air plasma‐treated PDMS is recovered after ≈24 h (Figure S1, Supporting Information).

**Figure 2:**
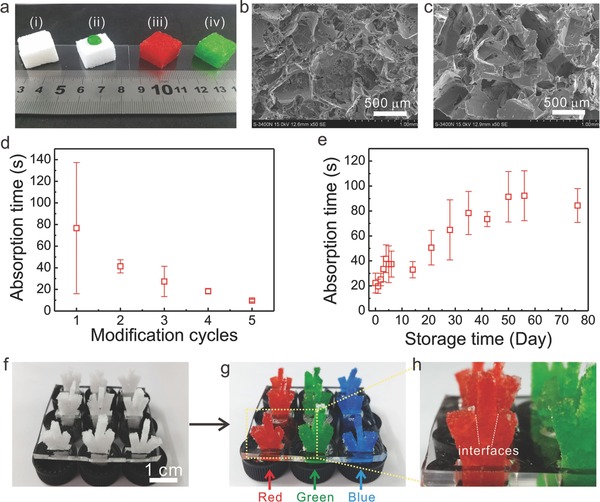
a‐i) Digital images of the original sugar cube, ii) PDMS sponge with one drop of green food dye solution on the top surface, iii) air‐plasma treated PDMS sponge absorbed with red food dye solution, and iv) PVA‐PDMS sponge absorbed with green food dye solution. SEM images of the as‐made PDMS sponges b) before and c) after surface modification with PVA solution. d) Relationship between the wettability (represented by the time for absorbing a 2 µL water droplet) and “dip‐coat‐dry” modification cycles. e) Hydrophilic stability test of the PVA modified PDMS sponges. Photograph of arrays of artificial trees made of the PVA‐PDMS sponges f) before and g) after absorption with food dye aqueous solutions. h) Magnified photograph of an artificial tree showing the interfaces between different sponges.

As a proof‐of‐concept demonstration, we fabricated arrays of artificial trees of PVA‐PDMS sponges (Figure 2f) and then applied them for the absorption of aqueous solutions containing x (red), y (green), z (blue) as dyes (Figure 2g). As shown in Figure 2h, the dye solution is ready to be absorbed even across the interfaces between different sponges, indicating the high wicking property of the sponges. The liquid phase was clearly observed by either naked eye or optical microscope (Figure S2, Supporting Information).

To demonstrate the power of PVA‐PDMS sponges as pumps, we applied the PVA‐PDMS sponges for laminar flow formation in a dandelion flower‐like microchannel (**Figure** **3**a). Six inlets at the “flower head” were connected to six different dye‐doped aqueous solutions separately through one piece of Teflon tubing. The outlet at the “flower stalk” was equipped with a PVA‐PDMS sponge pump. Once liquid filled up the microchannel and encountered the PVA‐PDMS sponge, the solutions from six baths were imbibed spontaneously and continuously into the microfluidic device driven by the PVA‐PDMS sponge pump. Laminar flow patterns were generated as shown in Figure 3a,b.

**Figure 3:**
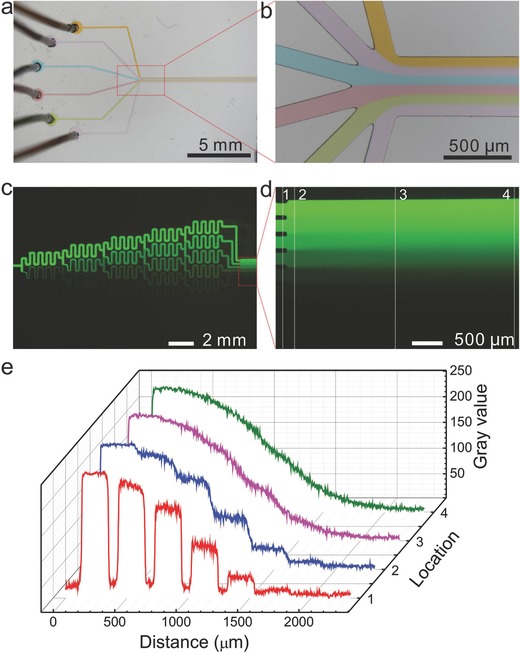
Microscopic images of the laminar flow a) formed in a dandelion flower‐like microchannel and b) its magnification. Fluorescent images of chemical gradient c) formed in a “Christmas tree” gradient generator, d) its magnified combined channel, and e) the grayscale plots representing four different locations indicated in (d).

Furthermore, the as‐made PVA‐PDMS sponge pumps are capable of actuating liquid displacement in even more complicated microchannel networks, such as the “Christmas tree” design gradient generator (Figure S3, Supporting Information). Similar to the previous procedures to create laminar flow, passive and self‐powered pumping by the PVA‐PDMS sponges was prompted when the contact between the sponge and solutions took place at the outlet. As shown in Figure 3c, a fluorescein solution (100 µmol L^−1^) and diethanolamine (DEA) buffer solution were absorbed into the network, respectively, and afterward the splitting and combining of mixed solutions happened repeatedly, leading to the well mixing in each serpentine channel. Seven terminal branches resulted in decreasing concentrations of fluorescein (from top to bottom) and generated a continuous concentration gradient in the downstream (Figure 3d,e). The produced chemical gradient is comparable to that powered by a syringe pump, revealing the excellent pumping capacity of PVA‐PDMS sponges.^36^

More importantly, dynamic manipulation of the flows was realized with the PVA‐PDMS sponges, which acted like leaves in a “non‐stop” pumping mode. As shown in **Figure** **4**a,b, by applying the sponge pumps, a good shape of laminar flow pattern was established within 3 min and maintained for more than 24 h. In fact, the pumping system still functioned well before the sponge was removed one day later, which demonstrated the possibility to exploit the PVA‐PDMS sponges for long duration applications in pumping. What is more, “stop‐flow” manipulation of the flow was adjusted via simply removing or applying the PVA‐PDMS sponges (Figure 4c). At stopped flow, which coincides with the removal of the sponge, the boundary of the laminar flow became blurry due to the diffusion of dye molecules across the interface. On the other hand, once returning the sponge, the flow started again and the laminar flow pattern was recovered in about half a minute. The separation and recombination of the sponge and the microchip did not affect the pumping performance and resulted in consistent flow patterns during the investigated three stop‐flow cycles (*vide* Figure 4c and Movie S1, Supporting Information). Based on this, a quasi‐stable high flow rate ranging from 6 to 15 µL min^−1^ was achieved by switching the loaded PVA‐PDMS sponge pump with unloaded (Figure 4d).

**Figure 4:**
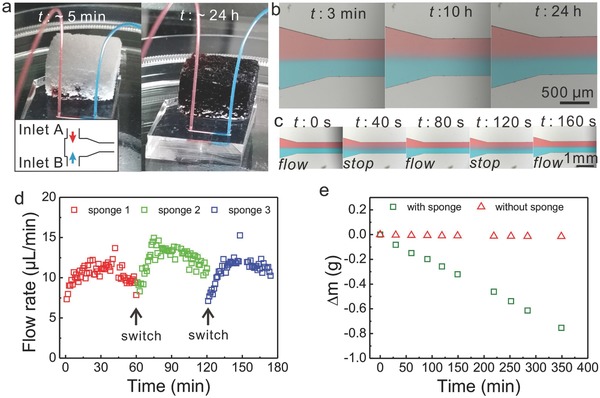
a) Digital images of the T‐junction microchip driven by a hydrophilic PVA‐PDMS sponge pump at the beginning (left) and after 24 h (right). b) Microscopic images of the laminar flow pattern generated in (a) at different time. c) “Stop‐flow” manipulation of the flows visualized by the breakdown and recovering of the laminar flow. d) Quasi‐stable high flow rates maintained by switching sponge pumps with unloaded ones at an interval of 1 h. e) Measurement of water evaporation rate with and without sponge pumps.

The tremendous absorption capacity of the as‐made PVA‐PDMS sponges can be attributed to (1) the capillary wicking and (2) evaporation of water at the sponge/air interface of the interconnected porous structures (Figure S4, Supporting Information). In the first step, liquids were driven passively by capillary forces and filled into the micropores embedded in the sponges. The increasing resistance because the empty pores were occupied gradually, which lowered the flow rate. This process lasted about 5 h and nearly 2 g of liquid were absorbed in total with a typical sponge presented here. Afterward, spontaneous water evaporation through the highly porous sponges dominated the actuation and refilled the micropores continuously. As shown in Figure 4e and Figure S5 (Supporting Information), the weight of the whole setup decreased linearly reaching a constant flow rate of 2 µL min^−1^ when the sponge pump was applied, while no obvious weight change was observed without the sponge.

In conclusion, we have demonstrated the fabrication and modification of durable hydrophilic PVA‐PDMS sponges by using a “dip‐coat‐dry” method and their application for bioinspired “non‐stop” pumping of gram scales of liquid. Without any external power sources, flows were generated continuously from the solution reservoir to the as‐made sponge owing to the capillary wicking and evaporation of water. This method has several advanced aspects, which are of significance to the development of liquid transport strategies and power‐free microfluidic devices, such as low‐cost/feasible fabrication process, long‐term durability of surface modification, manipulation of flows, and ultra‐long working duration. It is important to note that the “dip‐coat‐dry” modification preserves the presented sponges keeping their hydrophilicity more than 2 months. By applying the hydrophilic PVA‐PDMS sponges, both stable laminar flow patterns and chemical gradients in complex microchannel networks were realized, revealing the excellent pumping capacity of the hydrophilic sponges. What is more, even after the liquid fully loaded the sponge, the generated laminar flow still maintained a good shape during the investigated 24 h and appears to be “non‐stop” due to the coupled capillary/evaporation effects. Last and most importantly, quasi‐stable high flow rates above 6 µL min^−1^ with desired long‐term duration were achieved by simply switching the hydrophilic sponges. This intriguing feature should also be attributed to the “stop‐flow” manipulation of the flows via simple separation and merging of the sponge and the liquid at the outlet. The leaf‐like continuous water‐pumping strategy based on the hydrophilic 3D‐interconnected porous PDMS sponges is of significance to the better understanding of liquid transportation mechanism and further development of high‐performance devices for liquid delivery and flow manipulation. The techniques utilized in this work can be easily integrated to other miniaturized systems requiring pressure‐driven flow and may help to promote their use for extensive application areas, such as cell culture,^37^ micromixing,^38^ and continuous flow reaction,^39^ since the preparation of the presented hydrophilic sponges is inexpensive, facile and versatile, and not limited to PVA and PDMS.

## Conflict of Interest

The authors declare no conflict of interest.

## Supporting information

Supplementary

Supplementary
