# Supplementary material for: Hydrophilic Sponges for Leaf‐Inspired Continuous Pumping of Liquids
Source: Adv Sci (Weinh). 2017 Apr 19;4(6):1700028. doi: 10.1002/advs.201700028 (PMC5473324; doi:10.1002/advs.201700028)
Supplement: Supplementary file 1 — Supplementary [file ADVS-4-na-s001.pdf]

Copyright WILEY-VCH Verlag GmbH & Co. KGaA, 69469 Weinheim, Germany, 2017.

ADVANCED SCIENCE
Open Access

Supporting Information

for *Adv. Sci.,* DOI: 10.1002/advs.201700028

Hydrophilic Sponges for Leaf-Inspired Continuous Pumping of Liquids

*Tingjiao Zhou, Jinbin Yang, Deyong Zhu, Jieyao Zheng, Stephan Handschuh-Wang, Xiaohu Zhou, Junmin Zhang, Yizhen Liu, Zhou Liu, Chuanxin He, and Xuechang Zhou**

Copyright WILEY-VCH Verlag GmbH & Co. KGaA, 69469 Weinheim, Germany, 2013.

Supporting Information

Hydrophilic Sponges for Leaf-Inspired Continuous Pumping of Liquids

*Tingjiao Zhou,[1] Jinbin Yang,[1] Deyong Zhu,[1] Jieyao Zheng,[1] Stephan Handschuh-Wang,[1] Xiaohu Zhou,[1,2] Junmin Zhang,[1] Yizhen Liu,[1] Zhou Liu,[1] Chuanxin He,[1] and Xuechang Zhou[1,]**

1. T. J. Zhou, J. B. Yang, Dr. D. Y. Zhu, J. Y. Zheng, Dr. S. Handschuh-Wang, Dr. X. H. Zhou, Prof. J. M. Zhang, Dr. Y. Z. Liu, Dr. Z. Liu, Prof. C. X. He, Prof. X. C. Zhou,

College of Chemistry and Environmental Engineering,
Shenzhen University, Shenzhen, 518060, P. R. China
Fax: (+) 86-755-26536141; Tel: (+) 86-755-26536627
E-mail: xczhou@szu.edu.cn (X. C. Z.)

2. Dr. X. H. Zhou
Department of Chemistry, The Chinese University of Hong Kong,
Shatin, N.T., Hong Kong SAR, P. R. China

Chemicals and materials: Sugar cubes are commercial products and were bought from Tai Gang Food Co. Ltd (Shenzhen, China). The length, width, and height of the sugar cubes are 20.0 ± 0.5 mm, 18.0 ± 0.5 mm, and 10.0 ± 0.5 mm, respectively. Sylgard 184 was purchased from Dow Corning Corp. (Midland, MI, USA). Polyvinyl alcohol (PVA, Mw=130, 000) was purchased from Sigma-Aldrich. Food dyes were purchased from Gangdong Puning Shi Bao Food Co. Ltd (Puning, China). Fluorescein and Diethanolamine (DEA) were purchased from Macklin.

Solution preparation:

Preparation of 1 wt% PVA solutions. 0.5000 g PVA was added to 49.5000 g deionized water at 80 ^{o}C under stirring. Preparation of the food dye solutions. Red, blue, orange, purple, green,

pink food dyes were diluted at 20 times prior to use. Preparation of DEA buffer. 0.5257 g DEA was added to 500 mL deionized water under stirring, followed by addition of 1 $mmol \cdot L^{-1}$ $MgCl_2$ 1 mL. The DEA buffer was obtained after adjusting the pH value to 10.1 with 36% hydrochloric acid. Preparation of 100 $\mu mol \cdot L^{-1}$ fluorescein solution. 0.0033 g fluorescein was added to 100 mL DEA buffer under stirring. The obtained solution was filtrated by 0.45 μm PTFE filter prior to use.

Preparation of hydrophilic sponges: PDMS sponges were prepared using sugar cubes as templates (Figure 1). Sugar cubes were immersed in the mixture of PDMS prepolymer and curing agent at a weight ratio of 10:1. Then the sugar cubes in the mixture were degassed under vacuum for 2 h. PDMS on the surface of cubes was wiped off to expose the sugar after curing at 60 ^{o}C at atmospheric conditions for 3 h. The sugar template was then dissolved in hot water (60 ^{o}C) and washed away under stirring. After drying in a 60 ^{o}C oven for 10 h, PDMS sponges were obtained. To obtain hydrophilic PVA-PDMS sponges, the as-made PDMS sponges were fully activated with air plasma, followed by dipping in 1 wt% PVA solution for 20 min and further drying at 65 ^{o}C. After repeating the dip-coat-dry process for 5 times, the absorbed PVA at PDMS surface were heat-immobilized at 110 ^{o}C to produce hydrophilic PVA-PDMS sponges.

Preparation of artificial trees using the PVA-PDMS sponges: Artificial trees were assembled with the PVA-PDMS sponges as shown in Figure S2a. The artificial trees were immobilized by a PDMS holder and then were placed in small caps which were prefilled with red, green, blue food dye solutions. The artificial trees absorbed the solutions and turned to be colorful (Figure 2g-h).

Generation of laminar flow in T-junction and dandelion flower-like microchips: PDMS microchip and food dye solutions in centrifuge tubes, which were connected by Teflon tubings, were used to observe laminar flow. Microchannels were initially prefilled with solutions by using a syringe before applying the sponge pump. Then capillary action-driven pumping started to generate continuous flows when the hydrophilic sponge came into contact with the priming drop at the outlet.

Generation of chemical gradients in "Christmas tree" gradient generators: The design of the "Christmas tree" gradient generator is shown in Figure S3. The operation procedures for generating fluorescent gradient were similar as described in the previous part of laminar flow generation. The fluorescent images were taken by an excitation light of 460-500 nm.

Characterization methods: The morphology was verified with a scanning electron microscope (S-3400N, Hitachi, Japan) at an acceleration voltage of 15 keV. The contact angle and the water absorption time of hydrophilic PDMS sponge were measured by contact angle meter (SDC-200, Sindin, China). The volume of droplets in each measurement was 2 μL. All images of laminar flows and fluorescent gradients were recorded by the microscope (SMZ-18, Nikon, Japan) equipped with a CMOS camera (DS Ri-2, Nikon, Japan).

Flow rate measurement: The home-made flowmeter for measuring the pumping ability consisted of a microchip with the hydrophilic PVA-PDMS sponge pump and a syringe barrel (1 mL, inner diameter: 4.30 mm), which were connected by a piece of Teflon tubing (inner diameter: 300 μm). Images were taken at a time interval of 1 min using the microscope with a CMOS camera. By measuring the volume change of the green food dye solution in the syringe barrel, the flow rate in the microchannel powered by the sponge pump was calculated.

Evaporation rate measurement: As shown in Figure S5b-c, one flowmeter containing a prefilled PDMS sponge was used as the experimental group, while another one without sponge was the control group. The weight loss of the setups as the time change was measured by an analytical balance.

Movie S1: "Stop-flow" manipulation of the flows was visualized through breakdown and recovering of the laminar flow by simply removing or applying the hydrophilic PVA-PDMS sponges.

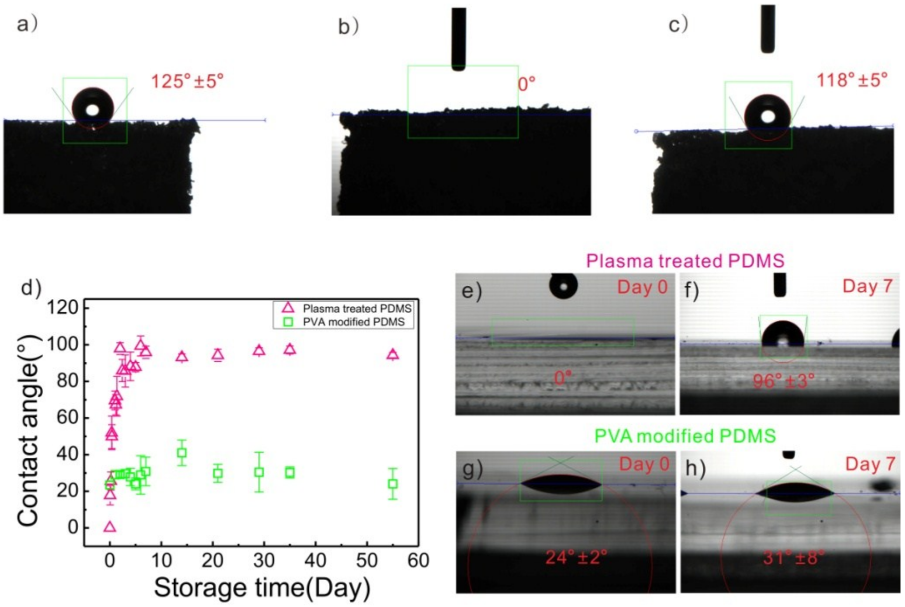

Figure S1. Hydrophilic stability of air-plasma treated or PVA modified PDMS. Photographs showing the contact angle on the surface of the original PDMS sponge (a), air-plasma treated PDMS sponge (b), and PDMS sponge with air-plasma treatment after 24 hours (c). (d) Hydrophilicity test of the air-plasma treated PDMS and PVA modified PDMS, and photographs showing the contact angle on the surface of the air-plasma treated PDMS at day 1 (e) and day 7 (f), and the PVA modified PDMS at day 1 (g) and day 7 (h).

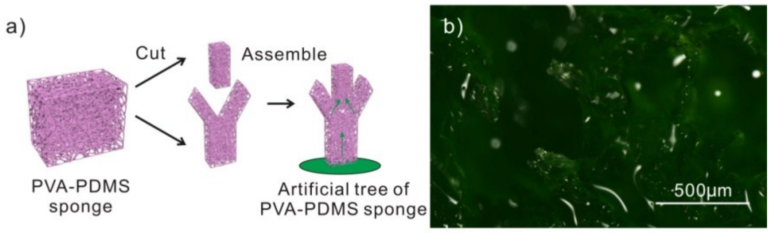

Figure S2. Arrays of artificial trees of PVA-PDMS sponges. (a) Schematic illustration of the fabrication of artificial trees using PVA-PDMS sponges. (b) Microphotograph showing the liquid phase absorbed into pores of the sponges.

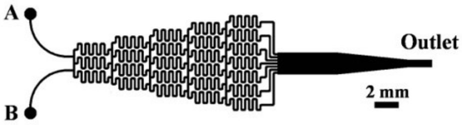

Figure S3. Design of the "Christmas tree" gradient generator. Fluorescein solution and DEA buffer solution were loaded through the inlet A and B, respectively.

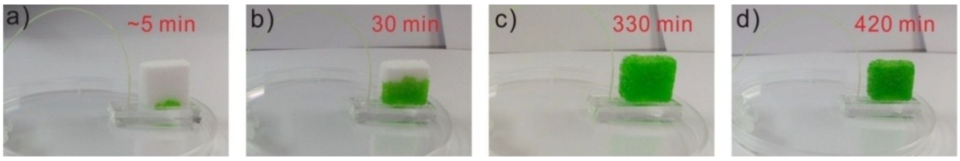

Figure S4. Liquid transport by the hydrophilic PVA-PDMS sponge. Digital images of the PVA-PDMS sponge at a different time: start of pumping (a), the half-filled state with a green solution (b), the initial state that full of green solution (c), and the final state with continuous flows driven by water evaporation (d).

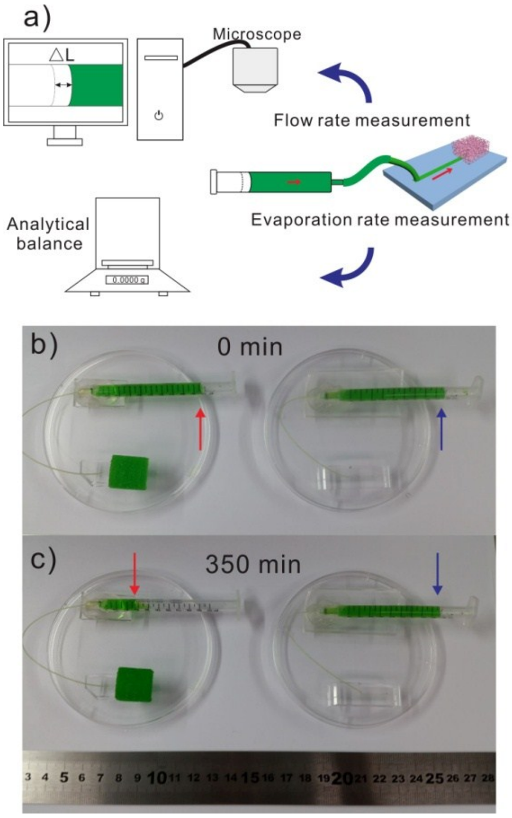

Figure S5. (a) Schematic illustration of methods for flow rate measurement and evaporation rate measurement. Digital images of the setups for water evaporation rate measurement with (left in both b and c) or without (right in both b and c) the hydrophilic PVA-PDMS sponge. Weights of the whole setups with or without the sponges were recorded at different time intervals. (temperature: 25 ± 0.5 ^{o}C, humidity: 53 ± 0.5 %)
